# Improved survival and MRD remission with blinatumomab vs. chemotherapy in children with first high-risk relapse B-ALL

**DOI:** 10.1038/s41375-022-01770-3

**Published:** 2022-12-08

**Authors:** Franco Locatelli, Gerhard Zugmaier, Carmelo Rizzari, Joan D. Morris, Bernd Gruhn, Thomas Klingebiel, Rosanna Parasole, Christin Linderkamp, Christian Flotho, Arnaud Petit, Concetta Micalizzi, Yi Zeng, Rajendra Desai, William N. Kormany, Cornelia Eckert, Anja Möricke, Mary Sartor, Ondrej Hrusak, Christina Peters, Vaskar Saha, Luciana Vinti, Arend von Stackelberg

**Affiliations:** 1 IRCCS Ospedale Pediatrico Bambino Gesù, Catholic University of the Sacred Heart, Rome, Italy; 2 Amgen Research (Munich) GmbH, Munich, Germany; 3 MBBM Foundation, University of Milano-Bicocca, Monza, Italy; 4 Amgen Inc., Thousand Oaks, CA USA; 5 Universitaetsklinikum Jena, Jena, Germany; 6 Universitätsklinikum Frankfurt am Main, Frankfurt, Germany; 7 Azienda Ospedaliera di Rilievo Nazionale Santobono Pausilipon, Napoli, Italy; 8 Medizinische Hochschule Hannover, Hannover, Germany; 9 Universitätsklinikum Freiburg, Freiburg, Germany; 10 Hopital Armand Trousseau, APHP.Sorbonne Université, Paris, France; 11 Istituto Pediatrico di Ricovero e Cura a Carattere Scientifico G Gaslini, Genova, Italy; 12 IQVIA Inc., Durham, NC USA; 13 Charité Campus Virchow-Klinikum Pädiatrie m.S. Onkologie/Hämatologie, Berlin, Germany; 14 Universitätsklinikum Schleswig-Holstein, Kiel, Germany; 15 Westmead Hospital, Sydney, NSW Australia; 16 Charles University Prague University Hospital, Prague, Czech Republic; 17 St. Anna Children’s Hospital, Children’s Cancer Research Institute, Vienna, Austria; 18 Division of Cancer Sciences, School of Medical Sciences, Faculty of Biology, Medicine and Health, University of Manchester, Manchester, UK; 19 Tata Translational Cancer Research Centre, Tata Medical Center, Kolkata, India; 20 Charité Universitaetsmedizin CVK Berlin, Berlin, Germany

**Keywords:** Drug development, Acute lymphocytic leukaemia

## To the Editor:

For children with high-risk, first-relapse B-cell precursor acute lymphoblastic leukemia (B-ALL), allogeneic hematopoietic stem cell transplantation (alloHSCT) after achieving a second complete remission (CR) remains the best, potentially curative treatment [1, 2]. In addition, a minimal residual disease (MRD)-negative status at the end of consolidation is an important prognostic indicator as demonstrated in the Children’s Oncology Group Studies AALL1131 (high risk) and AALL0932 (standard risk), and ALLR3 and ALL-REZ BFM 2002 studies [1, 3]. Blinatumomab, a CD3/CD19-directed bispecific T-cell engager (BiTE^®^) molecule, demonstrated a favorable benefit-risk profile prior to/after alloHSCT in patients with relapsed/refractory B-ALL in clinical trials and real-world experience studies [4–6], with early termination of enrollment in phase 3 trials in young adults and children because of blinatumomab benefit [4, 6]. In the phase 3 trial in pediatric high-risk, first-relapse B-ALL, blinatumomab consolidation pre-alloHSCT resulted in improved event-free survival (EFS) and MRD remission vs. chemotherapy, with EFS benefit consistently found in all subgroups, including those with extramedullary disease and very early relapse (<18 months) [6]. Enrollment was terminated for EFS benefit of blinatumomab (*p* < 0.001) per independent data monitoring committee’s (DMC) recommendation based on the July 2019 datacut. Follow-up data presented here are from September 2021, with overall survival (OS) benefit becoming apparent with longer follow-up.

## Methods

### Trial design

In this open-label, phase 3 trial (ClinicalTrials.gov: NCT02393859) [6], eligible patients were children with Philadelphia chromosome–negative, high-risk, first-relapse B-ALL post-induction and 2 consolidation cycles achieving an M1 (<5% blasts) or M2 (≥5% and <25% blasts) bone marrow [6]. Patients were randomized to receive either a third consolidation cycle with blinatumomab (15 μg/m^2^/day, 4 weeks, intravenous) or chemotherapy according to the IntReALL HR 2010 protocol [7]; children achieving/maintaining a second CR (M1 marrow) were eligible for alloHSCT. There were no restrictions on preparative regimen, donors, or stem cell source for alloHSCT.

The primary endpoint was EFS; events were relapse or M2 marrow post-CR, failure to achieve morphological CR at the end of treatment, second malignancy, or death (due to any cause), whichever occurred first. OS was the key secondary efficacy endpoint. Analysis was intent to treat, i.e., per randomization, including all patients for efficacy and those receiving protocol-specified therapy for safety. The trial protocol was approved by each center’s ethics committee/institutional review board. Parents or legal guardians provided written informed consent. Trial data were available to all authors and analyzed at Amgen.

## Results/discussion

Between November 2015 and August 2019, 111 patients were randomized (blinatumomab: 54, chemotherapy: 57) at 47 centers in 13 countries; data from the primary analysis with data cutoff of July 17, 2019, including 108 patients have been reported previously [6]. Enrollment was terminated for blinatumomab benefit (*p* < 0.001) per DMC’s recommendation (July 2019 analysis of ~1/2 of total EFS events). Data cutoff for this updated analysis clean snapshot was September 20, 2021. Baseline characteristics were comparable between the two randomization groups; notably, all patients randomized to blinatumomab received blinatumomab, while five patients randomized to chemotherapy did not receive it (Supplementary Tables 1 and 2).

**Fig. 1:**
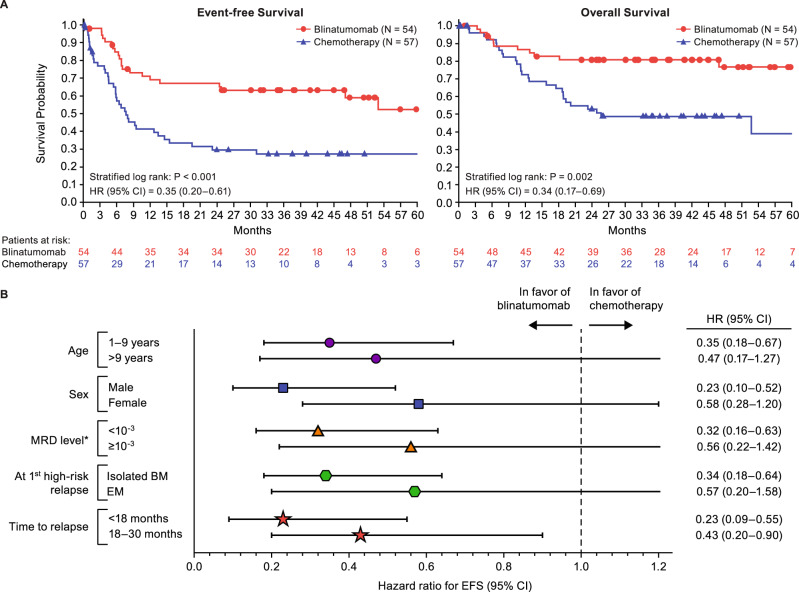
Survival by treatment arm and event-free survival subgroup analysis. **A** Survival probability over time is shown by treatment arm, i.e., blinatumomab or chemotherapy, for both event-free survival (left panel) and overall survival (right panel). Censoring indicated by circles and triangles for blinatumomab and chemotherapy, respectively. CI confidence interval, HR hazard ratio. **B** Event-free survival hazard ratios with 95% confidence intervals are shown for subgroups as indicated. *Stratification by marrow status (M1) at randomization and MRD after induction therapy. HR for M2 was not evaluable (*n* = 4 in each arm). BM bone marrow, CI confidence interval, EM extramedullary, EFS event-free survival, HR hazard ratio, MRD minimal residual disease.

After a median follow-up of 44 months, EFS was significantly improved with blinatumomab vs. chemotherapy with a hazard ratio (HR) of 0.35 (95% confidence interval (CI): 0.20–0.61, stratified log-rank *p* < 0.001, 4-year Kaplan–Meier estimates: 59% vs. 27%) (Fig. 1). EFS benefit was seen in all pre-specified subgroups, including very early relapse (i.e., <18 months from diagnosis) or extramedullary disease (probably due to better systemic control, although blinatumomab can cross the blood-brain barrier, and low concentrations of blinatumomab have been detected in the cerebrospinal fluid of patients with relapsed/refractory ALL [8]), and independent of baseline MRD (i.e., after 2 cycles of consolidation therapy). With longer follow-up, blinatumomab now demonstrates a strong benefit also for OS, with an HR of 0.34 (95% CI: 0.17–0.69, stratified log-rank *p* = 0.002, 4-year Kaplan–Meier estimates: 77% vs. 49%); previously, in the July 2019 primary analysis, the OS HR was 0.43 (95% CI: 0.18–1.01) [6]. EFS, OS, and MRD remission (<10^−4^ blasts by polymerase chain reaction (PCR), or flow-cytometry if PCR data were not available) were all improved with blinatumomab, both overall and independent of baseline MRD (< or ≥10^−3^) (Table 1), with >90% of MRD remissions achieved by day 15 [9]. This is particularly interesting, as day 15 MRD responses to blinatumomab in children with relapsed/refractory B-ALL were shown to predict response [10]. Further details on MRD response in the two randomization arms have been recently published [9]. A higher frequency of patients in the blinatumomab vs. chemotherapy arms proceeded to alloHSCT (94% vs. 68%).

**Table 1:** Outcomes by MRD status at baseline.

	Survival				MRD remission^b^	
	Blinatumomab	Chemotherapy	EFS^a^	OS^a^	Blinatumomab (*n* = 54)	Chemotherapy (*n* = 56)^c^
All	*N* = 54	*N* = 57	0.38 (0.22–0.65)	0.33 (0.17–0.68)	91 (80–97)	48 (35–62)
MRD < 10^−3^	*n* = 43	*n* = 37^c^	0.32 (0.16–0.63)	0.27 (0.10–0.68)	91 (78–97)	70 (53–84)
MRD ≥ 10^−3^	*n* = 11	*n* = 19^c^	0.56 (0.22–1.42)	0.63 (0.22–1.83)	91 (59–100)	5 (0.1–26)

*CI* confidence interval, *EFS* event-free survival, *HR* hazard ratio, *MRD* minimal residual disease, *OS* overall survival, *PCR* polymerase chain reaction.

^a^Data are unstratified HR (95% CI).

^b^Data are % response rate (95% CI) from cycle 1 day 29 of receiving study drug (i.e., blinatumomab or chemotherapy); MRD remission was defined as MRD < 10^−4^ by PCR, or flow-cytometry if PCR data were not available.

^c^One patient had inevaluable baseline MRD status.

Second relapses occurred in 17/54 (31%) blinatumomab and 35/57 (61%) chemotherapy patients (Supplementary Figs. 1 and 2). With blinatumomab, second relapses with extramedullary disease occurred in 6/54 (11%) patients, with central nervous system (CNS) involvement in 6 (11%); three patients had isolated extramedullary relapse (all solely CNS). With chemotherapy, second relapses with extramedullary disease occurred in 9/57 (16%) patients, with CNS involvement in 3 (5%); five patients had isolated extramedullary relapse (2 solely CNS). Late events (≥2 years on study) were all due to relapses. For the four blinatumomab patients with relapse 2–4.4 years on study, two had relapse in the CNS and two in the bone marrow; these relapses occurred 1.9–4.3 years post-alloHSCT. There was one chemotherapy patient with combined bone marrow and CNS relapse at 2.6 years on study and 2.5 years post-alloHSCT. CD19-negative relapse rates were low after both blinatumomab (2/54, 4%) and chemotherapy (1/57, 2%). CAR-T cell therapy after relapse post-study therapy was reported for 4/54 blinatumomab patients and 12/57 chemotherapy patients; 5 of the 12 chemotherapy patients received blinatumomab after developing relapsed/refractory disease and prior to CAR-T cells. In this small dataset, we did not observe evidence suggesting an adverse impact of blinatumomab on subsequent CAR-T cells.

Adverse events (AEs) were consistent with those reported in the primary analysis (Supplementary Table 3) [6]. No AE deaths were recorded. Grade ≥3 AE rates were 61% with blinatumomab and 83% with chemotherapy. There was no grade ≥3 cytokine release syndrome (CRS); CRS was reported in 1 chemotherapy patient (grade 1) and 2 blinatumomab patients (grade 1: 1, grade 2: 1). Grade ≥3 neurologic events were reported in 4 patients (blinatumomab: 3, chemotherapy: 1). In the blinatumomab arm, there were 2 patients with grade 3 events (neuropathic pain worsening to grade 3 following transplantation, unrelated to blinatumomab; treatment-related grade 3 dysphasia/depressed level of consciousness (study day 2) that resolved with blinatumomab discontinuation) and 1 patient with a grade 4 seizure (study days 2–3, resolved with blinatumomab discontinuation). In the chemotherapy arm, 1 patient had grade 3 confusion study days 3–5 attributed to ifosfamide, administration of which was interrupted.

Although there is heterogeneity in assessing CD19-negative relapse rates, when using treated patients as the denominator, low rates of CD19-negative relapse have been reported in blinatumomab trials of children [4, 5, 11] and adults [12, 13], with 1 analysis showing that reduced CD19 expression with blinatumomab was transient [14]. Other studies have shown that prior blinatumomab does not affect responses to CAR-T cells and vice versa [15]. Thus, the literature to date indicates that CD19 expression is preserved after blinatumomab treatment for the vast majority of patients who have relapsed, indicating that most would be eligible for subsequent anti-CD19 CAR-T cell therapy.

In conclusion, as compared with chemotherapy, treatment with blinatumomab for consolidation cycle 3 therapy pre-alloHSCT resulted in improved OS, EFS, and MRD remission rates, independent of baseline MRD, in children with high-risk first-relapse B-ALL. EFS benefit with blinatumomab was seen in all subgroups, including those with very early relapse and extramedullary disease. Extramedullary relapse rate was similar in blinatumomab and chemotherapy arms. The incidence of CD19-negative relapse was low in both treatment arms. The previously described better safety profile of blinatumomab treatment was confirmed with longer follow-up data. Whether earlier administration of blinatumomab therapy in the treatment course could provide additional benefit in this patient population remains to be explored.

## Supplementary information

Supplemental content

## Data Availability

Qualified researchers may request data from Amgen clinical studies. Complete details are available at the following: http://www.amgen.com/datasharing.
