# Supplementary material for: Improved survival and MRD remission with blinatumomab vs. chemotherapy in children with first high-risk relapse B-ALL
Source: Leukemia. 2022 Dec 8;37(1):222–5. doi: 10.1038/s41375-022-01770-3 (PMC9883152; doi:10.1038/s41375-022-01770-3)
Supplement: Supplementary file 1 — Supplemental content [file 41375_2022_1770_MOESM1_ESM.pdf]

Supplemental content

Methods

- Eligible patients had completed induction and the first 2 cycles of standard consolidation therapy, chosen from the IntReALL HR 2010, ALL-REZ BFM 2002, ALL R3, COOPRALL, and AIEOP ALL REC 2003 protocols at the investigator's discretion.[1-5]
- Stratification variables included age, bone marrow status after the second cycle of consolidation, and minimal residual disease (MRD) status after induction therapy.
- Blinatumomab was administered as 15 $\mu g/m^2$/day for 4 weeks by continuous intravenous infusion; dexamethasone (5 mg/m^2) was given before treatment on day 1 to prevent first-dose adverse events.
- Blinatumomab was discontinued for relapse, adverse event(s) requiring dose interruption at the 5 $\mu g/m^2$/day dose, clinically relevant toxicities that the investigator viewed as an unacceptable safety risk to the patient, clinically relevant neurologic events related to blinatumomab that required more than 1 week to resolve to grade ≤1, that were grade 3 or 4, or that occurred after restart of treatment, adverse events that did not resolve to grade ≤1 within 1 week or more than 2 interruptions per cycle due to adverse events, medical conditions that, in the opinion of the investigator, precluded further treatment, or withdrawal of patient's consent to further study treatment.
- Complete remission was defined as M1 marrow (≤5% blasts), no evidence of disease (i.e., peripheral blood without blasts and no extramedullary disease), and full recovery of peripheral blood counts.
- For allogeneic stem cell transplantation, there were no limits on preparative treatment (use of either total body irradiation or chemotherapy), donors (matched or mismatched siblings, haploidentical parents, matched or mismatched unrelated), or stem cell source (peripheral blood, bone marrow, or cord blood).
- MRD, defined as disease $<10^{-4}$, was assessed in parallel by multicolor flow-cytometry and real-time quantitative polymerase chain reaction (PCR) of clonal T-cell receptor or immunoglobulin gene rearrangements; if material was limited, only PCR was used.

- MRD status was checked at screening, day 15 (blinatumomab arm only), day 29, and post-allogeneic hematopoietic stem cell transplantation (alloHSCT) on 45 and 90 days and 6, 9, and 12 months. Survival outcomes and response rates were analyzed by pre-randomization MRD (i.e., after 2 cycles of consolidation therapy).
- Adverse events were graded per Common Terminology Criteria for Adverse Events v4.03 and were collected from the first dose of protocol-specified therapy through 30 days after the end of protocol-specified therapy or +90 days after alloHSCT.
- Time-to-event endpoints were summarized using the Kaplan-Meier method and treatment groups were compared using 2-sided stratified log-rank tests. A Cox regression model also tested for a treatment-by-subgroup interaction. Patients were censored at last disease assessment date if they ended the study prematurely or if still alive and event free.
- Treatment effects were described with a hazard ratio with 95% confidence interval (CI), estimated using a stratified Cox regression model. Percentages of patients with MRD remission were summarized with an exact binomial 95% CI.

Pharmacokinetic and antibody data

- For blinatumomab pharmacokinetic parameters, the mean [standard deviation (SD)] serum concentration at steady state was 921 (1010) pg/mL and mean (SD) clearance was 0.998 (0.450) L/h/m^2, with interpatient variability (i.e., percent coefficient of variation) in the parameter estimates of up to 109%. Given the high interpatient variability, mean (SD) blinatumomab steady-state concentration and clearance were generally within the range of those previously reported in pediatric patients from other blinatumomab studies.
- Forty-eight of the 54 (88.9%) patients in the blinatumomab arm had a post-baseline antibody result; none tested positive for binding or neutralizing anti-blinatumomab antibodies.

Supplemental Table 1. Demographics and baseline characteristics

	**Blinatumomab (N=54)**	**Chemotherapy (N=57)**
Age, median (range), years	6 (1-17)	5 (1-17)
1-9 years / >9 years, n (%)	39 (72) / 15 (28)	41 (72) / 16 (28)
Sex, male, n (%)	30 (56)	23 (40)
M1 bone marrow,* n (%)	54 (100)	54 (95)
MRD ($\geq 10^{-4}$ blasts),*,† n (%)	29 (54)	29 (51)
History of EM relapse at diagnosis of 1st high-risk relapse, n	10	15
Central nervous system	8	12
Genetic abnormalities at diagnosis of 1st high-risk relapse, n (%)		
Favorable prognosis	8 (15)	11 (19)
Hyperdiploidy	6 (11)	7 (12)
t(12;21)(p13;q22)/ETV6-RUNX1‡	2 (4)	4 (7)
Unfavorable prognosis§	7 (13)	9 (16)
t(v;11q23)/KMT2A rearranged	2 (4)	6 (11)
t(1;19)(q23;p13.3)/TCF3-PBX1‖	2 (4)	2 (4)
Hypodiploidy	2 (4)	0
Prognosis undefined	5 (9)	6 (11)

EM indicates extramedullary; MRD, minimal residual disease; PCR, polymerase chain reaction. *Per central laboratory assessment. †MRD at screening by PCR or flow cytometry. ‡Also known as TEL-AML1. §One patient in the blinatumomab arm with iAMP21 and 1 in the chemotherapy arm with t(17;19)(q22;p13)/TCF3-HLF also carried a genetic abnormality predicting an unfavorable prognosis. ‖Also known as E2A-PBX1.

Supplemental Table 2. Patient disposition

The full CONSORT diagram was previously published in Locatelli et al, *JAMA* 2021, 325(9):843-54.

	**Blinatumomab (N=54)**	**Chemotherapy (N=57)**
Patients randomized*	54 (100.0)	57 (100.0)
Received study regimen	54 (100.0)	52 (91.2)[†]
Completed study regimen	52 (96.3)	49 (86.0)
Discontinued study regimen	2 (3.7)	3 (5.3)
Adverse event	2 (3.7)	1 (1.8)[‡]
Required alternative therapy	0 (0.0)	2 (3.5)
Continuing study	38 (70.4)	20 (35.1)
Discontinued study	16 (29.6)	37 (64.9)
Death	10 (18.5)	27 (47.4)
Withdrawal of consent	4 (7.4)	9 (15.8)
Sponsor decision	2 (3.7)	1 (1.8)

All data are n (%). *Since July 2019, 3 patients already in the enrollment process were enrolled in the chemotherapy arm before the enrollment termination was completed. [†]Of the 5 patients in the chemotherapy arm who were randomized but did not receive study treatment, 4 withdrew consent and 1 died before receiving any study treatment. All 5 were included in efficacy analyses as intent-to-treat. [‡]The adverse event leading to discontinuation started before receiving study treatment.

Supplemental Table 3. Adverse events

	**Blinatumomab (N=54)**	**Chemotherapy (N=52)**
Fatal	0 (0.0)	0 (0.0)
Grade ≥3	33 (61.1)	43 (82.7)
Infections	25 (46.3)	18 (34.6)
Grade ≥3	11 (20.4)<br>7/11 post alloHSCT/ conditioning regimen	6 (11.5)<br>0/6 post alloHSCT/ conditioning regimen
Neurological	26 (48.1)	15 (28.8)
Grade ≥3*	3 (5.6)	1 (1.9)
Cytokine release syndrome	2 (3.7)	1 (1.9)
Grade 1	1 (1.9)	1 (1.9)
Grade 2	1 (1.9)	0 (0.0)

All data are n (%). AlloHSCT, allogeneic hematopoietic stem cell transplantation. *Grade ≥3 neurologic events observed with blinatumomab included 1 patient each with unrelated grade 3 worsened neuropathic pain starting on study day 54 following alloHSCT; serious treatment-related grade 3 dysphasia/depressed level of consciousness on study day 2, resolved with discontinuation of blinatumomab; serious treatment-related grade 4 seizures study days 2-3, resolved with discontinuation of blinatumomab. With chemotherapy, 1 patient had grade 3 confusion study days 3-5 attributed to ifosfamide, administration of which was interrupted.

Supplemental Figure 1. Relapse by treatment arm

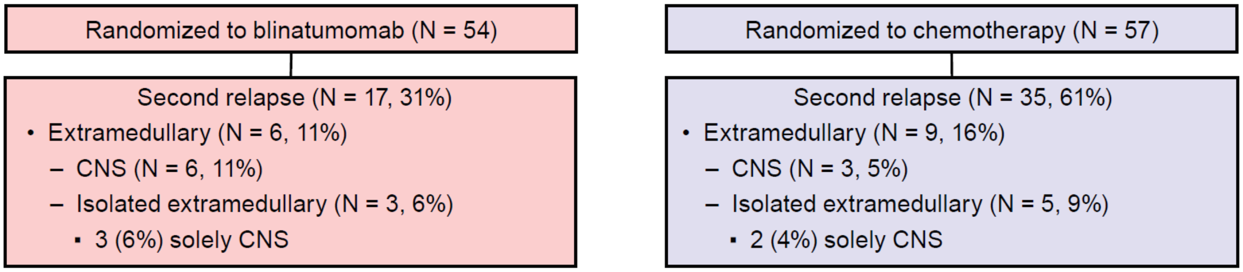

Relapse is shown by treatment arm (i.e., blinatumomab or chemotherapy), including those that are extramedullary and which are CNS. CNS, central nervous system.

Supplemental Figure 2. Cumulative incidence of relapse

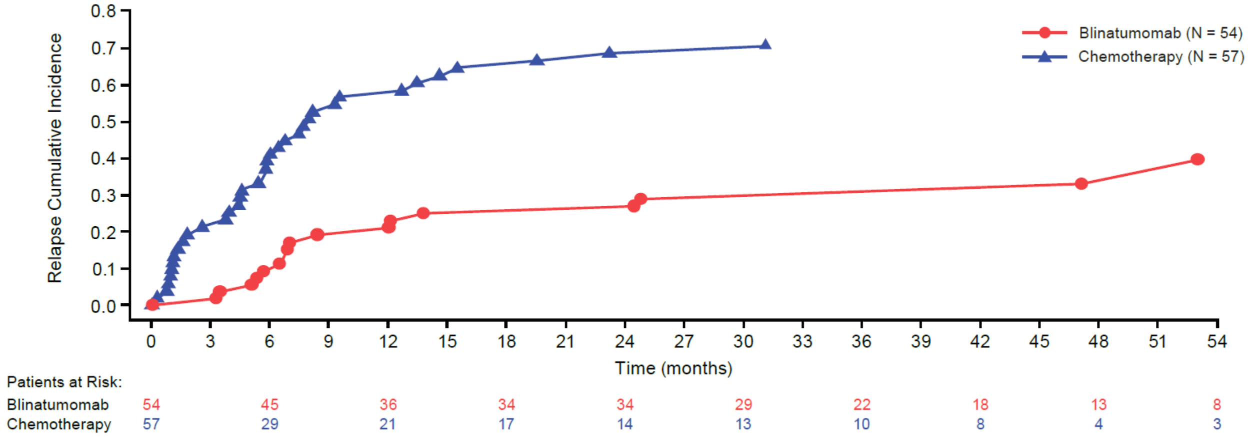

Incidence of relapse over time by treatment arm, blinatumomab or chemotherapy, is depicted.

References for supplemental data

1. Charité - University Hospital of Berlin. International Study for Treatment of Standard Risk Childhood Relapsed ALL 2010. EudraCT Number: 2012-000793-30. https://www.clinicaltrialsregister.eu/ctr-search/search?query=2012-000793-30.

2. Domenech C, Mercier M, Plouvier E, Puraveau M, Bordigoni P, Michel G, *et al*. First isolated extramedullary relapse in children with B-cell precursor acute lymphoblastic leukaemia: results of the Cooprall-97 study. Eur J Cancer. 2008; 44(16):2461–2469.

3. Eckert C, Henze G, Seeger K, Hagedorn N, Mann G, Panzer-Grümayer R, *et al*. Use of allogeneic hematopoietic stem-cell transplantation based on minimal residual disease response improves outcomes for children with relapsed acute lymphoblastic leukemia in the intermediate-risk group. J Clin Oncol. 2013; 31(21):2736–2742.

4. Paganin M, Zecca M, Fabbri G, Polato K, Biondi A, Rizzari C, *et al*. Minimal residual disease is an important predictive factor of outcome in children with relapsed 'high-risk' acute lymphoblastic leukemia. Leukemia. 2008; 22(12):2193–2200.

5. Parker C, Waters R, Leighton C, Hancock J, Sutton R, Moorman AV, *et al*. Effect of mitoxantrone on outcome of children with first relapse of acute lymphoblastic leukaemia (ALL R3): an open-label randomised trial. Lancet. 2010; 376(9757):2009–2017.
